# Virtual Induction Loops Based on Cooperative Vehicular Communications

**DOI:** 10.3390/s130201467

**Published:** 2013-01-24

**Authors:** Marco Gramaglia, Carlos J. Bernardos, Maria Calderon

**Affiliations:** 1 Institute IMDEA Networks, Avenida del Mar Mediterraneo 22, 28918 Leganes (Madrid), Spain; E-Mail: m.gramaglia@gmail.com; 2 Department of Telematics Engineering, Universidad Carlos III de Madrid, Avda. Universidad, 30, 28911 Leganes (Madrid), Spain; E-Mail: maria@it.uc3m.es

**Keywords:** vehicular communications, V2I, I2V, traffic monitoring

## Abstract

Induction loop detectors have become the most utilized sensors in traffic management systems. The gathered traffic data is used to improve traffic efficiency (*i.e.*, warning users about congested areas or planning new infrastructures). Despite their usefulness, their deployment and maintenance costs are expensive. Vehicular networks are an emerging technology that can support novel strategies for ubiquitous and more cost-effective traffic data gathering. In this article, we propose and evaluate VIL (Virtual Induction Loop), a simple and lightweight traffic monitoring system based on cooperative vehicular communications. The proposed solution has been experimentally evaluated through simulation using real vehicular traces.

## 1. Introduction

Every day, while we commute from home to work or from home to school, we likely traverse several detectors located along the roadsides that record our transit. Traffic data collected by these fixed sensors is used by public transport authorities (*i.e.*, city/regional/state) to improve traffic efficiency by, for example, warning users about accidents or congested areas, or planning new infrastructures. All this collected traffic data is processed by a central unit, which may decide to inform drivers about a potential event of interest.

The most common and reliable technology used to collect traffic data are induction loops [1]. These loops are embedded in roadways in a square formation that generates a magnetic field. Magnetic loops count the number of vehicles and collect some information for each vehicle traversing the loop, such as the instant of time, speed, lane and type of vehicle. This technology has been widely deployed all over the world in the last decades. However, the deployment and maintenance costs of the induction loops (ILs) are expensive [2].

Another existing monitoring technique is the use of video cameras. At the beginning cameras were only used for remote surveillance, but with the relatively recent improvements in image recognition and data analysis, video cameras are also currently being used to monitor road traffic load and state. Each vehicle is uniquely identified by its license plate number and then tracked over a defined stretch of the road. Although the use of image recognition tools can mimic the outcome obtained by induction loops [3], this still requires the deployment of specialized fixed-infrastructure (*i.e.*, camera posts). Moreover, video cameras might not properly operate at night or in severe weather conditions (e.g., fog, heavy rain or snow).

Vehicular networks based on short-range wireless communications are a new paradigm widely investigated nowadays to develop novel innovative Intelligent Transportation Systems (ITS) for safety and traffic efficiency. One-hop wireless communications among vehicles and, between vehicles and infrastructure nodes, enable the design of cooperative systems that can support novel decentralized strategies for ubiquitous and more cost-effective traffic data gathering.

In this context, this article proposes and evaluates VIL (Virtual Induction Loop), a simple traffic monitoring system based on cooperative vehicular communications. The basic idea behind VIL is to define virtual loops whose position is advertised by roadside units (RSUs) along the road. Vehicles, which are supposed to be equipped with Global Positioning System (GPS) receivers, monitor when they are traversing one of these virtual loops and store their state at that moment (e.g., time, speed and, lane). As soon as the vehicle gets close to an RSU, it delivers this recorded information to the RSU, which in turn collects information related to several vehicles and VILs and sends it to the central ITS station.

The resulting advantages from the deployment of VIL are manifold. More roads than those currently equipped with a monitoring infrastructure (mainly induction loops) could be easily observed without requiring additional costs (*i.e.*, nowadays only major urban cities can afford deploying and maintaining a monitoring infrastructure). VIL is a flexible and simple solution that incurs low communication overhead. VIL service is easily deployed using Context-Aware Messages (CAM) [4], standardized by the European Telecommunications Standards Institute (ETSI) to improve safety and traffic efficiency in roads. Furthermore, other foreseen ITS services may share the same communication infrastructure (e.g., pollution management).

The rest of this article is organized as follows. A brief review of ITS ETSI standardization is presented in Section 2. In Section 3 we detail our proposal, which is experimentally evaluated using a trace-driven simulator in Section 4, before concluding in Section 5.

## 2. Background

The ETSI Technical Committee for Intelligent Transport Systems ETSI TC ITS [5] is currently developing a set of protocols and algorithms that define a harmonized communication system for European ITS applications. Different types of ITS stations (e.g., vehicles) are defined [6], which have the capability of communicating between them using different access technologies. In particular, the IEEE 802.11p [7] at the 5.9 GHz band, an amendment to the 802.11 protocol especially tailored for vehicular networking, is one of these access technologies.

Vehicles can communicate with each other or with fixed roadside ITS stations (also called Roadside Units, RSUs) installed along roads. The roadside units, which are likely to be deployed uniformly along roads (e.g., using SOS posts), are usually connected to a wired network infrastructure (e.g., the Internet) and have a wireless interface to communicate with vehicles. Through the continuous exchange of messages between vehicles (Vehicle-to-Vehicle or V2V communications), and between vehicles and infrastructure nodes (Vehicle-to-Infrastructure or V2I communications), real-time information about current road traffic conditions can be cooperatively collected and shared.

According to ETSI standards, ITS stations, vehicles and RSUs periodically broadcast secure Cooperative Awareness Messages (CAM) [4] to neighboring ITS stations that are located within a single hop distance. CAMs are distributed using 802.11p and provide information of presence, positions as well as basic state of communicating ITS stations (e.g., current acceleration, occupancy of the vehicle, current heading of the vehicle, …). The periodic exchange of CAMs helps ITS stations to support higher layer protocols and cooperative applications, including road safety and traffic efficiency applications.

## 3. VIL Operation

A virtual induction loop (VIL) is a virtual line playing the same role as a legacy magnetic induction loop (IL). In this way, VIL service gathers real time information of the vehicles traversing this virtual line.

**Figure 1.**
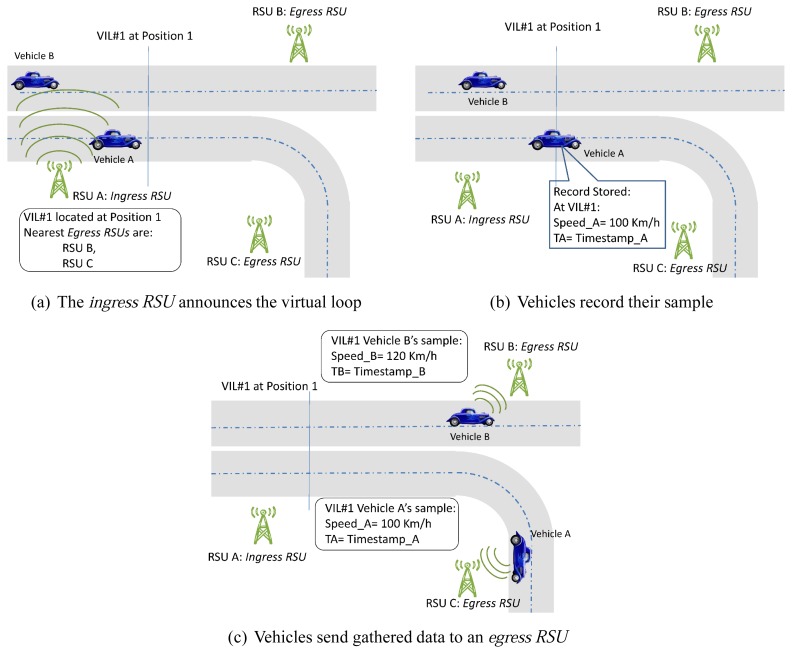
VIL operation.

VIL is a traffic efficiency service that makes use of existing secure CAM messages sent by RSUs and vehicles [4]. The full operation is shown in Figure 1. First, the ingress RSU announces in its CAM messages the positions of the virtual induction loops present in the stretch of road under its influence. These CAM messages also include information on the identities of the egress RSUs, that is, the nearest RSUs the vehicle may find in its way, after traversing the virtual loops in this stretch, depending on the vehicle's trajectory (see Figure 1(a)). It is important to highlight two details: first, each stretch can not only have several egress RSUs, but also several ingress RSUs (*i.e.*, announcing the same virtual loops); second, an RSU may play the role of egress RSUs for a stretch and the role of ingress RSUs for the next stretch.

On the other hand, GPS devices are now cheap enough to be included in vehicles configurations, as part of the on-board computer system. They supply two valuable pieces of information: the current position and the current time in Universal Time Coordinated (UTC) form. From these two variables, it is straightforward to calculate the current speed as well.

Thus, for each announced virtual loop a vehicle encounters during its transit within the monitored stretch (see Figure 1(b)), it records its state when traversing the virtual loop (e.g., timestamp, speed, lane, *etc.*).

At some point, the vehicle becomes aware of being within the radio coverage of an egress *RSU* upon the reception of a CAM message broadcast by the egress *RSU* (*i.e.*, the CAM message includes the identity of this egress *RSU*). From that moment on, and while being in the RSU's coverage area, CAM messages sent by the vehicle also include the information gathered when it traversed each of the virtual loops in the last stretch (see Figure 1(c)). In addition to the basic information (e.g., timestamp and speed), the vehicles can also upload another useful data such as the lane or their characteristics (e.g., traffic control centers often want to know the percentage of heavy lorries). The *egress RSUs* send the data gathered to the traffic control center. Once there, the information on traffic conditions is elaborated and eventually redistributed to drivers.

### 3.1. Impact of Technology Penetration Rate

Section 4 provides an evaluation of our solution, showing no significant differences between the data collected by using real induction loops and the one obtained from our virtual induction loop solution. So far, we have presented our solution assuming an ideal penetration rate situation, meaning that every vehicle on the road is equipped with a VIL-enabled ETSI TC ITS device. However, virtual induction loops can be deployed even in non-ideal scenarios in which not all the vehicles are VIL-enabled or have any communication capabilities.

**Figure 2.**
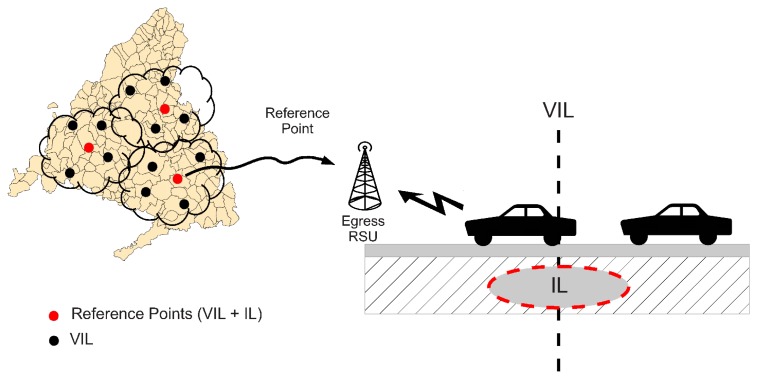
Reference factor estimation.

If VIL is operated in a scenario where not all the vehicles participate in the virtual sensing, the estimated information will deviate from the real one. In order to correct this behavior, VIL can use a reference factor accounting for the ratio of vehicles that are VIL-enabled over the total of vehicles traversing a particular region. This can be easily achieved by using real induction loops that are already deployed to obtain reference values of the number of vehicles using a road, and then crossing that information with the number of VIL-enabled vehicles measured by a virtual induction loop placed at the same position of the real loop, as shown in Figure 2. Since the number of real induction loops is lower than the intended number of virtual loops, the reference value is obtained by averaging the information gathered from closest neighboring loops (e.g., loops located in the same city or the same metropolitan area). The time granularity used to estimate this reference factor can also be adjusted depending on the road characteristics. A similar correction factor, taking into account market penetration statistics, was also proposed by Bauza *et al.* in [8].

It is worth noting that even with lower penetration rates, VIL can provide an equivalent service to real induction loops, but as the number of VIL-equipped vehicles increases, VIL could also be used to enable more complex services, such as traffic congestion prediction.

## 4. Evaluation

We evaluated our proposal by simulation. To achieve realistic results we built our software using the Veins framework [9] for OMNeT++ [10]. Veins [11] is an advanced vehicular communication simulator that couples realistic wireless features with the microscopic mobility simulator SUMO [12].

**Figure 3.**
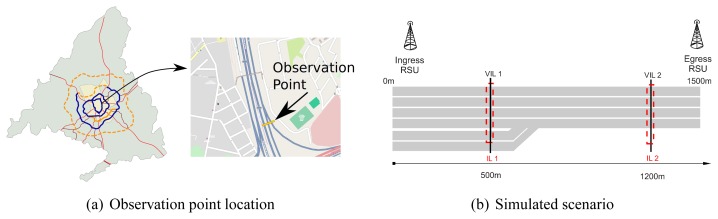
Real data and simulated environment.

The goal of our simulator is to realistically investigate the effectiveness of VIL. To this aim, we consider both realistic wireless conditions and realistic vehicular patterns. To achieve the second goal we fed our simulator using real vehicular traces captured by magnetic induction loops. The data was kindly provided to us by the Madrid City Council and was collected along the M-30 orbital motorway (see Figure 3(a)). The trace is 2.5 hour long (from 8:00 AM to 10:30 AM) and it contains data of about 17,000 vehicles. A sample of the used data is shown in Table 1.

In our experiments the refresh frequency of the GPS is *f_r_* = 1*Hz* (update frequency of position and current time), which is an additional source of error in the experiments. However, this refresh frequency is considered realistic taking into account the features of the current commercial GPS devices.

Aiming at introducing realistic noise to the experiment, we introduce a Gaussian white noise for the two position components (both latitude and longitude) with a parameter *σ* = 4*m*, which is a fair error assumption for highways under clear conditions. The main simulation parameters are summarized in Table 2.

The monitored stretch is 1,500 m long and it is a 6-lane single carriageway road, composed by 4 main lanes and 2 acceleration lanes. In the stretch there are one ingress *RSUs*, one egress *RSUs* and two virtual loops (see Figure 3(b)).

**Figure 4.**
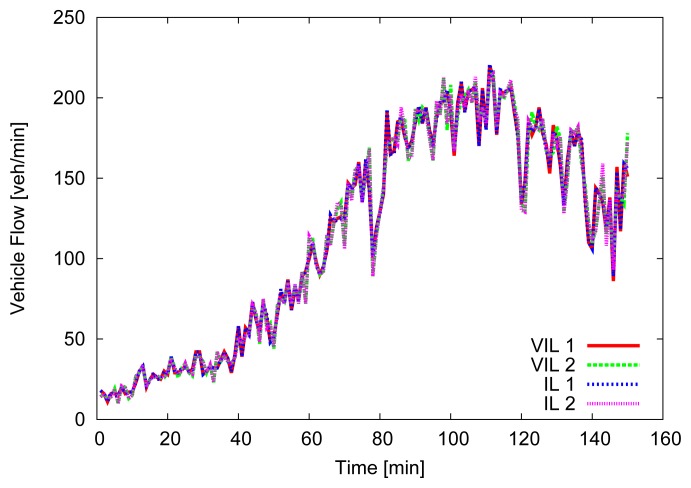
Vehicular flow.

**Figure 5.**
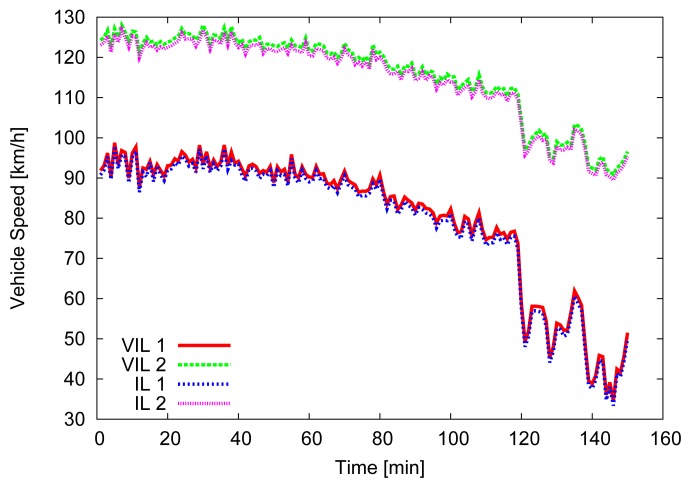
Average speed.

**Figure 6.**
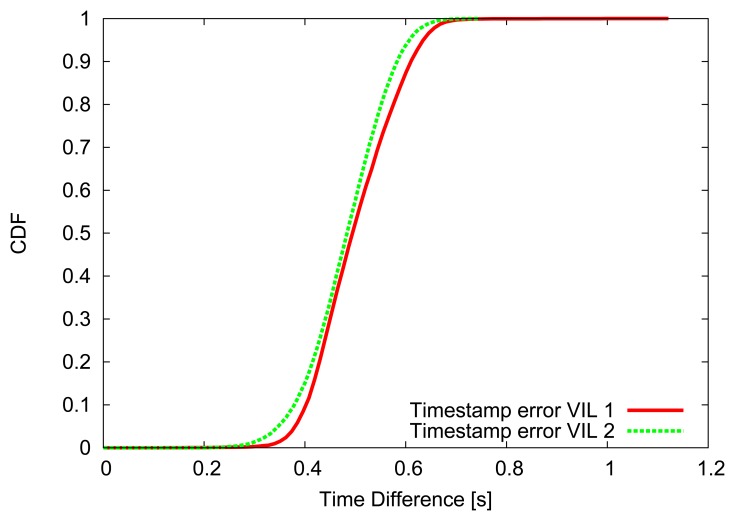
Crossing timestamps error.

For the first 500 m, where the first VIL is placed, vehicles' speed is forced to be close (±2.5 m/s) to the departure one, then vehicles are allowed to increase their speed, up to 130 km/h. Figures 4, 5 and 6 depict the obtained simulation results. As it is typically done, the vehicular metrics—flow and average speed—are summarized into coarser bins, in our case one-minute sized. In Figures 4 and 5, we compare the flow and the mean speed obtained by VIL at the egress RSU with the real ones from the vehicular traces obtained with real induction loops. It can be noticed that despite inaccuracy in latitude and longitude, and the errors introduced by the refresh frequency, the gap between the two curves is almost negligible. Figure 6 shows the cumulative distribution function (CDF) of the difference (in absolute value) between the real crossing timestamps and the ones measured by the VIL system. As it can be observed, almost all the crossing timestamps obtained by VIL fall into ±0.8 s of difference with the real ones.

A relevant parameter to be taken into account is the control overhead that the system introduces over the air. Due to the use of existing CAMs, no new messages are introduced by VIL system. However, the size of CAM messages is increased with the VIL information. In particular, CAMs broadcast by ingress RSU include the location of the virtual loops in the area under their influence (8 bytes per virtual loop) and the identity of the nearest egress RSUs (8 bytes per RSU). Considering the case of a CAM message that announces 3 virtual loops and 5 egress RSUs, the total increase in the CAM message size is 64 bytes. As for the CAM messages sent by the vehicles when they are in the coverage area of an egress RSU, the size of these CAMs is increased by 6 bytes (the timestamp field accounts for 4 additional bytes, and the speed one for 2 more bytes) for each virtual loop the vehicle has crossed. Therefore, the increase in the CAM message size can be considered to be of negligible impact.

## 5. Conclusions

Keeping the traffic conditions monitored is a crucial task in traffic management systems. Traditional solutions, based on the use of magnetic induction loops, are widely deployed, even though they incur high installation and maintenance costs. In this article we propose a simple approach based on cooperative vehicular communications that allows scalable monitoring without incurring high installation costs. Simulation results show that both the introduced error compared with the legacy methodologies and the wireless overhead are negligible. Even in initial deployment scenarios, where not every vehicle is VIL-enabled, our solution can be used as a suitable replacement of legacy monitoring systems. Moreover, VIL enables a configurable level of granularity, providing a higher data resolution just when and where it is needed.

## Figures and Tables

**Table 1.** Trace sample.

Timestamp	Vehicle #	Lane #	Speed (km/h)
08:57:00:6	5831	4	59
08:57:00:8	5832	6	61
08:57:00:9	5833	3	49
08:57:01:2	5834	4	68
08:57:01:7	5835	6	61
08:57:01:8	5836	1	68
08:57:01:8	5837	2	48

**Table 2.** Simulation settings.

Simulation framework	OMNeT++, Veins and SUMO
Wireless Device	802.11g @ 6 Mb/s
Channel Model	Pathloss with channel fading
Monitored stretch length (m)	1500
VIL positions (from the *ingress RSU*) (m)	500, 1000
CAM frequency (s)	uniform (0.75,1.25)
